# The safety and efficacy of percutaneous intraductal radiofrequency ablation in unresectable malignant biliary obstruction: A single-institution experience

**DOI:** 10.1186/s12885-017-3278-5

**Published:** 2017-04-24

**Authors:** Wei Cui, Wenzhe Fan, Mingjian Lu, Yingqiang Zhang, Wang Yao, Jiaping Li, Yu Wang

**Affiliations:** Department of Interventional Oncology, the First Affiliated Hospital, Sun Yat-sen University, Guangzhou, 510080 China

**Keywords:** Radiofrequency catheter ablation, Self expandable metal stent, Bile duct obstruction, Radiology, interventional

## Abstract

**Background:**

Patients with unresectable malignant biliary obstruction have limited life expectancy because of limited stent patency and tumor progression. The aim of our study was to retrospectively evaluate the safety and efficacy of combining intraductal RFA with biliary metal stent placement for patients with malignant biliary obstruction.

**Methods:**

Patients who received percutaneous intraductal RFA and biliary stent placement for malignant biliary obstruction between 2013 and 2015 were identified. Outcomes were stent patency, technique and clinical success rate, overall survival (OS) and complication rates. Kaplan-Meier and Cox regression analyses were used to examine the association of various factors with stent patency and OS. Complications and laboratory abnormalities were recorded.

**Results:**

Fifty patients were treated with percutaneous RFA and stent placement. The rates of technical success and clinical success were 98% and 92%, respectively. The median stent patency was 7.0 (95% confidence interval [CI]: 5.3, 8.7) months and OS was 5.0 (95% CI: 4.0, 6.0) months. On univariable analysis, previously cholangitis was an independent poor prognosis factor for recurrent biliary obstruction. OS was improved in patients who received more than one intervention compared to those who received only one intervention (log-rank *P* = 0.007), and in those treated without versus those treated with sequential chemotherapy (log-rank *P* = 0.017). On multivariable analysis, the occurrence of more than one intervention (*P* = 0.019) had independent prognostic significance for OS.

**Conclusion:**

Percutaneous RFA and stent placement is a technically safe and feasible therapeutic option for the palliative treatment of malignant biliary obstruction. The long-term efficacy and safety of the procedure is promising, but further study is required via randomized and prospective trials.

## Background

Patients with malignant biliary obstruction due to different types of tumors, including pancreatic, bile duct, gallbladder, and hepatocellular carcinomas, frequently have a poor prognosis in terms of quality of life and survival. These cancers are often surgically unresectable at the time of diagnosis, and those that are resected have high recurrence rates [1]. Placement of self-expandable metal stents (SEMS) is the standard of care in the palliative management of patients with malignant biliary strictures if their life expectancy is at least 3 months [2]. However, maintaining patency is a problem, with tumor overgrowth, epithelial hyperplasia, biofilm deposition, and sludge formation limiting the median patency of metal stents to a mere 6 months [3].

Despite attempts to find a potential solution to the problem of SEMS occlusion, including the use of covered stents, different stent designs, and biliary intraluminal irradiation stents, little progress has been made in terms of improving the duration of stent patency [4–7]. Although photodynamic therapy (PDT) has been reported as an alternative capable of increasing the rate of stent patency, it is expensive and time consuming, and is associated with cholangitis and photosensitivity [8, 9]. Recently, a Percutaneous Endobiliary Radiofrequency catheter (Habib™ PERF; EMcision Ltd., London, UK) was developed specifically to attempt to solve the problem of stent occlusion. Studies using ex-vivo and in-vivo pig models have clarified the effectiveness of the Habib PERF catheter, with an output power of 7–10 W and an ablation time of 2 min recommended for clinical treatment [10, 11]. An open-label pilot study involving 22 patients with malignant biliary strictures confirmed the safety and feasibility of this radiofrequency ablation (RFA) technique for clinical use [12]. The technique has shown promising results in the palliative treatment of malignant biliary strictures, preventing stent occlusion [13–18], clearing blocked metal stents [7], prolonging stent patency [19], and improving patient survival [20]. We previously reported our early experience in managing patients with unresectable Bismuth types III and IV hilar cholangiocarcinoma using biliary RFA, and demonstrated that the long-term efficacy and safety is promising [21]. Here, we describe a broader experience in managing 50 patients using biliary RFA in an effort to improve long-term stent patency in unresectable malignant biliary obstruction. We also examined the prognostic factors for stent patency in these patients.

## Methods

We conducted a retrospective study of consecutive patients with malignant biliary obstruction who underwent biliary RFA and stent placement at the First Affiliated Hospital of Sun Yat-sen University between 2013 and 2015. Malignant biliary obstruction was diagnosed in all cases on the basis of the characteristic clinical features (jaundice and/or clay colored stool), laboratory tests (elevated bilirubin levels and alkaline phosphatase levels), and imaging findings. Access to the database and the methods used for data retrieval and analysis were approved by the ethics committee of our hospital, and written informed consent was obtained from each participant in accordance with the Declaration of Helsinki.

### Study participants

The inclusion criteria were (1) age ≥ 20 years; (2) malignant biliary obstruction confirmed using computed tomography (CT) or abdominal magnetic resonance imaging (MRI), with pathological confirmation whenever possible; (3) clinical jaundice, a serum bilirubin level greater than 5 mg/dL, and/or cholangitis; (4) performance status score ≤ 2 [22]; (5) unresectability or refusal to be surgically treated. Eligible patients were those with biliary obstruction due to cancer of the pancreas, gallbladder, or bile ducts; primary and secondary liver cancers; or regional lymph node metastases, who were considered unsuitable for surgery because of distant metastases, vascular invasion, or severe disability due to age or associated diseases. Non-resectability was established through the consensus opinion of a multidisciplinary tumor board. Identified patients were screened with the following exclusion criteria: (1) performance status score ≥ 3; (2) identification of a secondary malignancy; and (3) lost to follow-up or missing data.

### Treatment

The Habib™ EndoHPB is an 8-Fr. (2.6 mm), 1.8-m long bipolar RFA catheter with two radiologically marked electrodes at its tip and is inserted over a 0.035-in. guide wire into the bile duct [21]. This catheter can be used for either an endoscopic retrograde cholangiopancreatography (ERCP) or a percutaneous transhepatic cholangiography (PTC) procedure. Under digital subtraction angiography (DSA) guidance, PTC was performed to localize the site of biliary obstruction and to confirm its length and diameter. A guide wire was then passed through the stenosis via the percutaneous drainage catheter. The Habib EndoHBP probe was advanced over the wire with the tip of the probe placed across the malignant stricture. The probe was attached to a standard high-frequency generator, with 10 W applied for 90 s [21]. For patients with long segmental obstruction of the bile duct, RFA was performed section by section. For patients with high-level obstruction and tumors involving the bilateral bile ducts, RFA of the bilateral intrahepatic bile ducts was necessary. Immediately after RFA, uncovered SEMS (Wallstent; Boston Scientific, Boston), mounted on a delivery system, were placed. Generally, the SEMS were selected according to the individual radiologist’s preference and the manufacturer’s protocol [21]. Cholangiography was used to confirm that the bile duct was clear. Follow-up assessment of drainage flow was performed under DSA guidance three to four days after the procedure, and the catheter was removed if the flow remained unobstructed.

### Re-intervention

In the event of recurrence of cholangitis or jaundice, abdominal CT was performed to verify stent patency, and when dilation of the drained bile duct was confirmed, repeat-ablation with or without a stent was attempted. On the other hand, when dilation of the drained bile duct was not confirmed, focal cholangitis of another undrained branch of the bile duct was suspected, and stent placement with RFA was attempted for that branch. If drainage failed, percutaneous transhepatic cholangial drainage was performed.

### Assessment and follow-up

Technical success was defined as passage of the stent across the stricture, with good radiographic positioning, along with flow of contrast and/or bile through the stent [23]. Clinical success was defined as the improvement of symptoms such as jaundice, pruritus, and total bilirubin levels to less than half or less than the normal upper limit within 14 days. Stent patency was defined as the interval between the first stent insertion procedure and the recurrence of the symptoms of restructure without repeat-ablation or stent insertion. If there was no evidence of obstruction while the patient was alive, the patency period was considered to be equal to the survival period, but was censored. Stent patency was confirmed by the absence of jaundice, normal levels of direct bilirubin, and the absence of bile duct expansion on US, CT or MR imaging during follow-up [19]. Overall survival (OS) was calculated from the date of the first procedure until the date of death.

The incidence of complications associated with the procedures was investigated. The major adverse events that were assessed included bleeding, infection, pancreatitis, pain, recurrent biliary obstruction, and bile perforation. Mild bleeding was defined as no requirement for transfusion within 48 h. Moderate bleeding was defined as a need for a blood transfusion of more than 2 units or a haemostatic procedure, including both pharmaceutical and surgical intervention, after a drainage procedure [24]. Acute pancreatitis was diagnosed in the presence of elevated pancreatic enzyme levels ≥3 times the upper limit of the normal range within 24 h of the procedure and with symptoms of pancreatitis. Post-procedure pain was defined as follows: (1) mild pain, which was noted as any pain requiring short-term treatment with oral analgesics, (2) moderate or severe pain, which included any symptoms necessitating hospital admission or the use of intravenous analgesics [25]. The definitions of causes of recurrent biliary obstruction, such as tumor ingrowth or overgrowth, and stent migration, were based on the 2014 Tokyo criteria for transpapillary biliary stenting [26].

Outcomes were stent patency, OS, technical success, clinical success, and complications. After adequate palliation of the biliary obstruction, patients were discharged, with follow-up arranged through the outpatient clinic at two-week to three-month intervals. Patients’ continuing medical history and the results of physical examination and laboratory studies were included in the medical record. Patients who died were excluded at the date of their last follow-up. Follow-up continued from the first operation to the death of the patient or the end of the study.

### Statistical analysis

Descriptive statistics were calculated, using the mean ± standard deviation (SD) or median and range, as appropriate for the data type. Stent patency and OS were evaluated using Kaplan-Meier curves, with between-group differences compared using the log-rank test. Variables with potential prognostic significance for stent patency and OS were assessed through univariable analysis. Significant variables on univariable analysis were included in a multivariable Cox regression model. All analyses were performed using SPSS statistics software (SPSS, Version 16.0 for Windows; Chicago, IL). All *P* values were two sides, with a level of 0.05 considered to be significant.

## Results

### Patient characteristics

In the final analysis, 50 patients who received intraductal RFA and stent placement for unresectable malignant biliary obstruction between 2013 and 2015 were included. The baseline characteristics are shown in Table 1. Among the patients, 38% (*n* = 19) had undergone prior primary tumor resection, 22 (44%) had cholangitis, and 29 (58%) had distant metastases at baseline. The mean baseline total and direct bilirubin (TB, DB) levels were 198.4 μmol/L (median, 168 μmol/L; SD, 167.2 μmol/L) and 108.1 μmol/L (median, 95.1 μmol/L; SD, 83.4 μmol/L), respectively. The mean baseline gamma-glutamyl transpeptidase level (GGT) was 405.68 U/L (median, 311 U/L; SD, 278.2 U/L).

**Table 1:** Patient Characteristics

Category	Subcategory	Number (%)
Total		50
Median age(range), yr.		61.8(41–85)
Sex		
	Male	36(72)
	Female	14(28)
Type of tumor		
	Pancreatic carcinoma	10(20)
	Gallbladder carcinoma	4(8)
	Cholangiocarcinoma	25(50)
	Hepatocelluar carcinoma	6(12)
	Lymph node metastases	5(10)
Level of biliary obstruction^*^		
	Common bile or hepatic duct (type I)	11(22)
	Type II	8(16)
	Type III A	10(20)
	Type III B	4(8)
	Intrahepatic (type IV)	17(34)
Performance status score		
	0	10(20)
	1	20(40)
	2	18(36)
Previously cholangitis		22(44)
Distant Metastasis		29(58)
No. of interventions, mean (range)		1.2(1–3)
Subsequent chemotherapy		7(14)

Note. — Unless otherwise indicated, data are number of patients and data in parentheses are percentages

^*^According to the Bismuth classification of perihilar cholangiocarcinoma

### Treatment details

All patients received percutaneous intra-ductal RFA and stent placement, and 14% (*n* = 7) received subsequent platinum-based chemotherapy. Unilateral stent placement was performed in 39 (78%) patients, with 11 (22%) patients requiring bilateral stents at the initial procedure. Forty-two (84%) patients underwent one ablation and stent placement session, while six (12%) underwent two sessions (four of them without new stent placement), and two (4%) underwent three ablations without stent placement sessions due to recurrent biliary obstruction.

### Outcomes

Complications related to the procedures are shown in Table 2. No severe complications, such as bile duct perforation, bile leak, or acute pancreatitis, were identified post-procedure. Four patients required blood transfusion for post-procedure bleeding. However, two patients died within 30 days after the RFA procedure, both due to cholangitis and septic shock. Furthermore, one patient with a history of coronary heart disease, percutaneous coronary intervention, atrial fibrillation, hypertension, and hyperthyroidism, developed an acute state of chronic heart failure caused by atrial fibrillation and rapid ventricular rate. Conservative treatment was successful for this patient. Of note is the incidence rate of new cholangitis, with an overall rate of 32% (16 of 50 patients). Patients presented symptoms of bacterial cholangitis, with antibiotic treatment being successful to resolve fever and normalize white blood cell counts.

**Table 2:** Outcome of procedures in two groups

Outcome	Number (%)
Mild bleeding	23(46)
Moderate bleeding	4(8)
Mild pain	18(36)
Moderate or severe pain	4(8)
Bile infection	16(32)
Acute pancreatitis	0
Recurrent obstruction	
Tumor ingrowth	6(12)
Tumor overgrowth	8(16)
Stent migration	1(2)
New stricture	3(6)
Unkown	1(2)

**Fig. 1:**
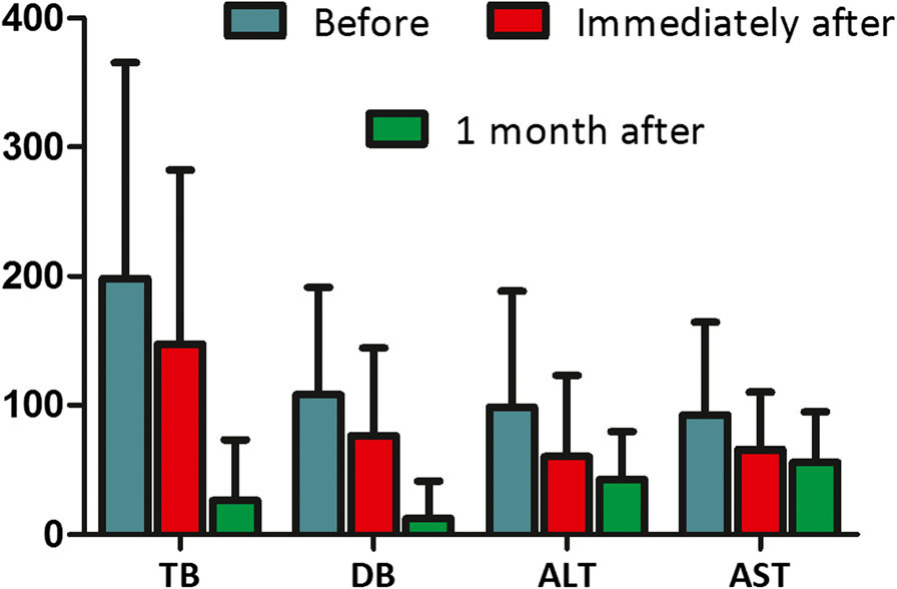
Liver function before and after RFA and stent placement. Bar chart shows the results of liver function tests before and after RFA and stent placement. Total bilirubin (TB), direct bilirubin (DB), alanine aminotransferase (ALT), and aspartate aminotransferase (AST) were obtained before, immediately after, and 1 month after RFA and stent placement. Data are means ± standard errors of the mean

The rates of technical and clinical success were 98% (*n* = 49) and 92% (*n* = 46). Liver function tests were performed before, immediately after (2–4 days after the procedure), and 1 month after the procedure in all patients except for the two who died within 30 days (Fig. 1). Between the time before and the time immediately after ablation, the following parameters improved significantly: mean TB (P < 0.001), DB (P < 0.001), alanine aminotransferase (ALT) (P < 0.001), and aspartate aminotransferase (AST) (P < 0.001). Short-term follow-up showed the preservation of increased liver function for 1 month.

**Fig. 2:**
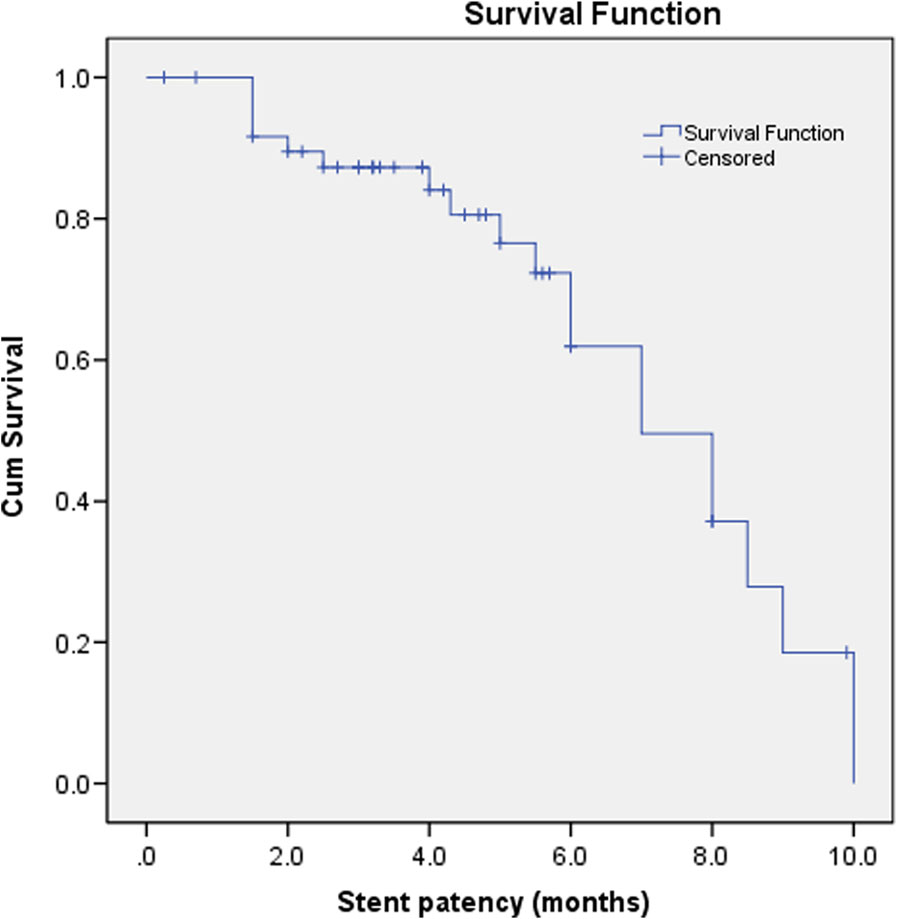
Kaplan-Meier curve of stent patency. The calculation started on the day of the first RFA procedure and extended to the time of proven stent occlusion, stent migration, or patient death

**Fig. 3:**
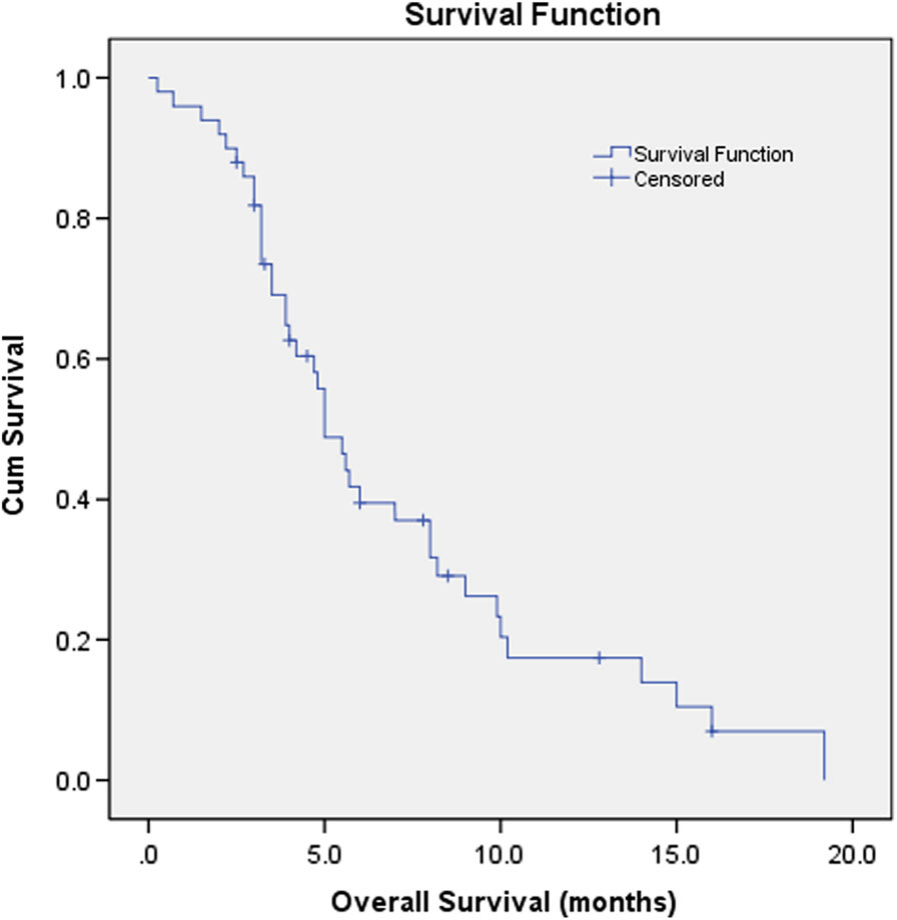
Kaplan-Meier survival curve of overall survival. The calculation started on the date of the first procedure until the date of death or last follow-up

The median follow-up was 6 months, and 10 (20%) patients were still alive at the time of data analysis. Five patients died of recurrent cholangitis and sepsis shock, one of heart disease, two of gastrointestinal haemorrhage, and 32 of tumor progression. The median stent patency was 7.0 (range 1.5–10, 95% confidence interval [CI]: 5.3, 8.7) months and median survival (from the first procedure until death or last follow-up) was 5.0 (range 0.25–19.2, 95% CI: 4.0, 6.0) months (Figs. 2 and 3). Univariable and multivariable Cox regression analyses for factors associated with stent patency and OS are presented in Table 3 and Table 4. In univariable analysis, there was no significant difference in the stent patency when patients were stratified by age, sex, performance status score, level of biliary obstruction, distant metastasis, or sequential chemotherapy (*P* = 0.024). Previously cholangitis was an independent poor prognosis factor for recurrent biliary obstruction (Table 3). However, OS was improved in patients who received more than one intervention compared to those who received only one intervention (*P* = 0.007), and in those treated without versus those treated with sequential chemotherapy (log-rank *P* = 0.017). On multivariable analysis, only the occurrence of more than one intervention remained independently significant (Table 4). None of the tested cut-off points for TB, DB, ALT, AST, and GGT were statistically significant in univariable and multivariable analyses (Table 3 and Table 4).

**Table 3:** Risk of Recurrence of Biliary Obstruction in Patients with Unresectable Malignant Biliary Obstruction after Therapy

		Univariate Analysis		Multivariate Analysis	
Variable*	No. of cases	HR(95%CI)	P†	HR(95%CI)	P^†^
Age(≥62 y vs < 62 y)	25/25	2.348(0.840,6.566)	0.104		
Sex(male vs female)	36/14	1.243(0.404,3.827)	0.705		
Tumor diagnosis (Cholangiocarcinoma vs other neoplasms)	25/25	1.770(0.679,4.612)	0.243		
Type of obstruction(proximal vs distal)	19/31	0.899(0.324,2.494)	0.838		
Performance status score (2 vs ≤1)	32/18	1.706(0.890,3.270)	0.108		
Previously cholangeitis (no vs yes)	28/22	3.347(1.176,9.525)	0.024	3.347(1.176,9.525)	0.024
Distant Metastasis(yes vs no)	29/21	1.292(0.490,3.403)	0.605		
Chemotherapy (no vs yes)	43/7	0.840(0.277,2.547)	0.759		
TB(≥168.0 μmol/L vs <168.0 μmol/L)	25/25	0.488(0.184,1.294)	0.149		
DB(≥95.1 μmol/L vs < 95.1 μmol/L)	25/25	0.485(0.183,1.287)	0.146		
ALT (≥71 U/L vs <71 U/L)	25/25	1.352(0.491,3.728)	0.560		
AST (≥72.5 U/L vs < 72.5 U/L)	25/25	0.887(0.348,2.261)	0.801		
GGT(≥311 U/L vs < 311 U/L)	25/25	0.613(0.228,1.652)	0.334		

Note—*TB* = total bilirubin, *DB* = direct bilirubin, *ALT* = alanine aminotransferase, *AST* = aspartate aminotransferase, *G-GT* = gamma-glutamyl transpeptidase

*For continuous variables, cutoff level chosen for continuous variables was their median value

† *P* values were determined with Cox proportional hazards regression models. *P* < 0 .05 indicated a significant difference

**Table 4:** Risk of Death in Patients with Unresectable Malignant Biliary Obstruction after Therapy

		Univariate Analysis		Multivariate Analysis	
Variable*	No. of cases	HR(95%CI)	P†	HR(95%CI)	P^†^
Age(≥62 y vs < 62 y)	25/25	0.776(0.379,1.589)	0.488		
Sex(male vs female)	36/14	0.803(0.475,1.359)	0.414		
Tumor diagnosis (Cholangiocarcinoma vs other neoplasms)	25/25	0.825(0.439,1.552)	0.552		
Type of obstruction(proximal vs distal)	19/31	0.932(0.490,1.775)	0.825		
Performance status score (2 vs ≤1)	32/18	1.706(0.890,3.270)	0.108		
No. of interventions (1 vs ≥2)	42/8	0.187(0.055,0.630)	0.007	0.295(0.106,0.818)	0.019
Previously cholangeitis (no vs yes)	28/22	0.794(0.405,1.555)	0.500		
Distant Metastasis(yes vs no)	29/21	0.897(0.474,1.698)	0.739		
Chemotherapy (no vs yes)	43/7	0.273(0.094,0.792)	0.017	2.0.465 (0.159,1.365)	0.164
TB(≥168.0 μmol/L vs <168.0 μmol/L)	25/25	1.038(0.544,1.978)	0.911		
DB(≥95.1 μmol/L vs < 95.1 μmol/L)	25/25	0.901(0.475,1.706)	0.748		
ALT (≥71 U/L vs <71 U/L)	25/25	1.495(0.791,2.825)	0.216		
AST (≥72.5 U/L vs < 72.5 U/L)	25/25	1.087(0.575,2.056)	0.798		
GGT(≥311 U/L vs < 311 U/L)	25/25	1.100(0.584,2.072)	0.767		

Note—*TB* = total bilirubin, *DB* = direct bilirubin, *ALT* = alanine aminotransferase, *AST* = aspartate aminotransferase, *G-GT* = gamma-glutamyl transpeptidase

*For continuous variables, cutoff level chosen for continuous variables was their median value

† *P* values were determined with Cox proportional hazards regression models. *P* < 0 .05 indicated a significant difference

## Discussion

We report the efficacy and complications encountered at our institution for 50 patients treated with percutaneous intra-ductal RFA for unresectable malignant biliary obstruction. Compared to the 3 to 6-month patency of metal stents alone, the median stent patency of 7.0 months observed in this study is promising and highlights the urgent need for the use of the procedure in select patients [3]. Until now, PDT was the only evidence-based treatment other than stenting that improved the quality of life and survival of such patients [27]. Recently, Daniel [25] reported that RFA and PDT are associated with comparable survival in the treatment of unresectable cholangiocarcinoma. Therefore, these studies all suggest a possible survival benefit in the use of RFA. However, the results must be interpreted cautiously, because of the retrospective design with many sources of bias. The improvements of SEMS patency, cost-effectiveness, and survival advantages should be confirmed by further randomized controlled trials, if any. The main limitations of our study included its retrospective nature, the fact that it was a single-center experience with heterogeneous etiologies, and lack of a control group.

The potential therapeutic effect of combined RFA and stent placement has only been evaluated in a few studies to date, with mixed findings reported [9, 19, 20, 28, 29]. The 7-month stent patency reported here (95% CI: 5.3, 8.7) is in agreement with the results of the study reported by Werner et al. (median metal stent patency: 7.3 months) [29] but is higher than that reported by Tian-Tian et al. (stent patency: 5.0 months) [28]. Our study and that reported by Werner et al. had higher rates of cholangiocarcinoma (50% and 88%, respectively) and sequential chemotherapy (14% and 41%, respectively) compared with the rates in the trial reported by Tian-Tian et al. (48% and 4%, respectively), which is a possible explanation for this discrepancy. Therapeutically, intraluminal RFA and stent placement offers several benefits. Foremost, the local thermal effect can destroy the malignant biliary stricture, resulting in a local coagulation necrosis that has the potential to delay tumor growth and, therefore, prolong the duration of stent patency [16]. This local effect on tumor tissue was confirmed by Monga et al. [13], who reported the disappearance of tumor blood vessels and enlargement of the lumen following RFA. Moreover, RFA can be used to clear the occlusion of a previously deployed metal stent, and restore the biliary flow without the need to insert a new stent inside the obstructed stent [7]. As the use of RFA is not restricted to a single session, repeated treatments and multiple sessions can be performed to increase tumor destruction and improve the stent patency, which is an independent prognosis factor for patient survival (Table 4). Successful biliary drainage and long-term stent patency are important to alleviate clinical symptoms, confer some benefits to improve liver function (Fig. 1), improve performance status scores, and provide an opportunity for subsequent treatment of the primary tumor. However, the median OS (5.0 months) is shorter than the stent patency (7.0 months). This is likely because many patients still had patent stents at the time of the last follow-up or death. Furthermore, due to local tumor progression, many patients in our study died of disease progression but not recurrent stent obstruction, and only a few patients received repeat-ablation. In the Cox analysis, OS was improved in patients who received more than one intervention compared to those who received only one intervention (log-rank *P* = 0.007). The RFA heat penetration depth is limited and the coagulation zone is too small in patients who have tumors that involve the bile duct but do not originate from the bile duct epithelium (such as pancreatic cancer, enlarged hilar node). Therefore, the indications of intra-ductal RFA remain controversial, and the basic theory of this technique as a palliative treatment in extra-luminal biliary compression should also be further studied. Additionally, previous studies mostly utilized the endoscopic approach with this technique for the treatment of malignant biliary obstruction. Whether the endoscopic approach is better than the percutaneous route warrants further study.

Considering that covered SEMS are associated with increased risk of pancreatitis and cholecystitis but have comparable benefits to uncovered stents, uncovered SEMS placement was performed for all patients in our study. There were no severe complications, such as bile duct perforation, bile leak, or acute pancreatitis. However, we observed atrial fibrillation and sudden onset of chronic heart failure in one patient after the procedure, which might be a relative contraindication for this technique. We do emphasize that the incidence of cholangitis (32%) was very high in our group and two patients died within 30 days after the RFA procedure, both due to cholangitis and septic shock. Accordingly, we fully support the prophylactic administration of antibiotics before stent placement to lower the risk of cholangitis [19, 20]. Additionally, in univariable analysis, previously cholangitis was an independent poor prognosis factor for recurrent biliary obstruction (Table 3).

## Conclusions

In conclusion, percutaneous RFA and stent placement is a technically safe and feasible therapeutic option for the palliative treatment of malignant biliary obstruction. We present more of our experiences with the use of this technique for select patients, and demonstrate the feasibility, with a satisfactory safety profile.

## Abbreviations

ALT: alanine aminotransferase
AST: aspartate aminotransferase
DB: direct bilirubin
DSA: digital subtraction angiography
ERCP: endoscopic retrograde cholangiopancreatography
GGT: gamma-glutamyl transpeptidase
OS: overall survival
PDT: photodynamic therapy
PTC: percutaneous transhepatic cholangiography
RFA: radiofrequency ablation
SEMS: Self-expandable metal stents
TB: total bilirubin
